# Dynamics of Planktonic Prokaryotes and Dissolved Carbon in a Subtropical Coastal Lake

**DOI:** 10.3389/fmicb.2013.00071

**Published:** 2013-04-08

**Authors:** Maria Luiza S. Fontes, Denise Tonetta, Larissa Dalpaz, Regina V. Antônio, Maurício M. Petrucio

**Affiliations:** ^1^Programa de Pós-graduação em Ecologia, Departamento de Ecologia e Zoologia, Universidade Federal de Santa CatarinaFlorianópolis, Santa Catarina, Brazil; ^2^Graduação em Ciências Biológicas, Departamento de Ecologia e Zoologia, Universidade Federal de Santa CatarinaFlorianópolis, Santa Catarina, Brazil

**Keywords:** planktonic prokaryotes, carbon dioxide, subtropical lake, DOC, cyanobacteria

## Abstract

To understand the dynamics of planktonic prokaryotes in a subtropical lake and its relationship with carbon, we conducted water sampling through four 48-h periods in Peri Lake for 1 year. Planktonic prokaryotes were characterized by the abundance and biomass of heterotrophic bacteria (HB) and of cyanobacteria (coccoid and filamentous cells). During all samplings, we measured wind speed, water temperature (WT), pH, dissolved oxygen (DO), precipitation, dissolved organic carbon (DOC), dissolved inorganic carbon (DIC), and carbon dioxide (CO_2_). DOC was higher in the summer (average = 465 μM – WT = 27°C) and lower in the winter (average = 235 μM – WT = 17°C), with no significant variability throughout the daily cycles. CO_2_ concentrations presented a different pattern, with a minimum in the warm waters of the summer period (8.31 μM) and a maximum in the spring (37.13 μM). Daily trends were observed for pH, DO, WT, and CO_2_. At an annual scale, both biological and physical-chemical controls were important regulators of CO_2_. HB abundance and biomass were higher in the winter sampling (5.60 × 10^9^ cells L^−1^ and 20.83 μmol C L^−1^) and lower in the summer (1.87 × 10^9^ cells L^−1^ and 3.95 μmol C L^−1^). Filamentous cyanobacteria (0.23 × 10^8^–0.68 × 10^8^ filaments L^−1^) produced up to 167.16 μmol C L^−1^ as biomass (during the warmer period), whereas coccoid cyanobacteria contributed only 0.38 μmol C L^−1^. Precipitation, temperature, and the biomass of HB were the main regulators of CO_2_ concentrations. Temperature had a negative effect on the concentration of CO_2_, which may be indirectly attributed to high heterotroph activity in the autumn and winter periods. DOC was positively correlated with the abundance of total cyanobacteria and negatively with HB. Thus, planktonic prokaryotes have played an important role in the dynamics of both dissolved inorganic and organic carbon in the lake.

## Introduction

Lakes are sentinels of global change to the extent that their storage and transformation of organic matter changes with global warming, with which there are many concerns (Cole et al., 2007; Adrian et al., 2009; Tranvik et al., 2009). Lakes generally function as sources of carbon dioxide (CO_2_) to the atmosphere, mainly because of high inputs to and degradation through mineralization of terrestrial organic material in lakes (Cole et al., 1994, 2007; Kosten et al., 2010; Marotta et al., 2010a,b). However, CO_2_ emissions among lakes vary, with tropical lakes responsible for a significant portion of CO_2_ emissions compared to temperate lakes (Marotta et al., 2009a,b; Kosten et al., 2010). In addition, annual variability of these factors in lakes has also been reported (Trolle et al., 2012). Despite the studies on temporal and spatial variability of carbon fluxes among and within temperate and tropical lakes, little is known about the temporal dynamics in subtropical lakes and what factors drive carbon variability there.

The temperature dependence of community respiration and thus metabolic rates (Kosten et al., 2010; Yvon-Durocher et al., 2012), and of cyanobacteria dominance in shallow lakes (Kosten et al., 2012; Sarmento, 2012) suggest that in tropical and subtropical lakes, prokaryotes play a major role in carbon mineralization and production (via primary production) (Sarmento, 2012).

Studies encompassing the daily variability of planktonic prokaryotes and carbon are rare. A few authors have described bacterial abundance and daily shifts in respiration (Pringault et al., 2007, 2009; Sadro et al., 2011), but to our knowledge, none have investigated bacterial biomass variability and its relationship with dissolved carbon on daily and annual scales simultaneously.

Thus, our main objectives were to evaluate the dynamics of planktonic prokaryotes at two time scales (48 h and annual), including their relationship with dissolved inorganic and organic carbon in a subtropical lake.

## Materials and Methods

### Study site

**Figure 1:**
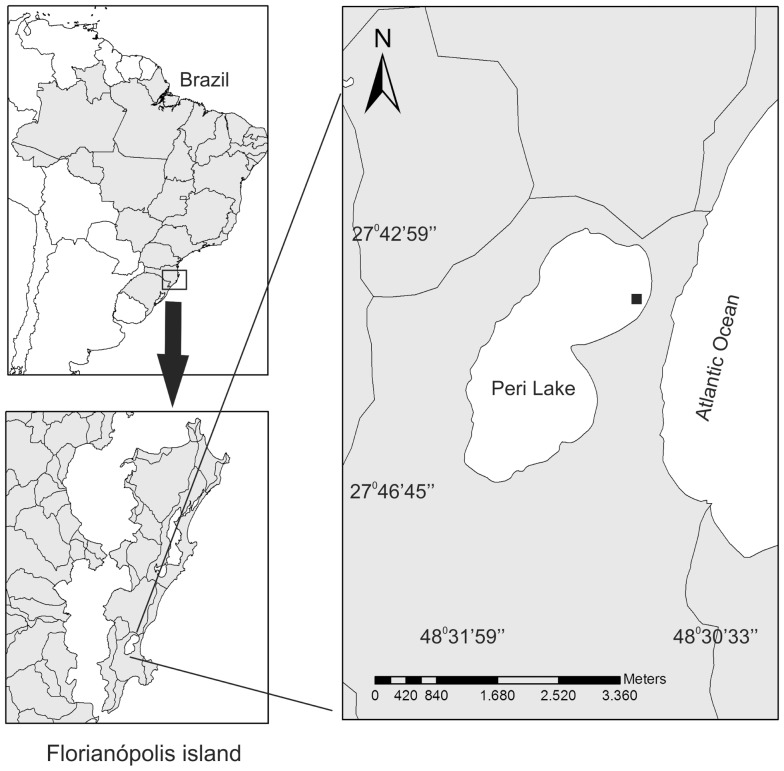
**Map of Peri Lake, showing the location of the sampling site (black dot) in the littoral zone**.

The study site is located in the littoral zone of the Peri coastal lake (Figure 1). The lake is surrounded by Atlantic Rain Forest and is separated from the sea by a vegetated sandbank, which isolates Peri Lake and prevents exchange of water with the adjacent ocean. Consequently, it is a freshwater system. Peri Lake is located in a protected area called “Parque Municipal da Lagoa do Peri (PMLP)” and supplies potable water for a significant percentage of the population of Florianópolis. It has a surface area of 5.7 km^2^, a maximum length of 4 km, an average width of 1.7 km, and average and maximum depths of 4.2 and 11 m, respectively. There are two main tributaries of the lake, which contain a volume of 21.2 million cubic meters of water. Peri Lake is classified as oligotrophic for nutrients and mesotrophic for chlorophyll-*a* (Chl-*a*) (Hennemann and Petrucio, 2011). The sampling site was located in the littoral zone of the lake (Figure 1), where the depth oscillates between 0.5 and 0.6 m deep, and the water column is continuously mixed.

### Meteorological variables

Wind velocity and precipitation were obtained over 9 days (7-days prior to and during the 48-h samplings) from EPAGRI/CIRAM (Centro de Informações de Recursos Ambientais e de Hidrometeorologia de Santa Catarina), which has a station near the lake.

### Experimental description

Aliquots of water were collected from the site at 3-h intervals during daylight and at 6-h intervals at night in October/2009, March/2010, May/2010, and August/2010 (representing austral spring, summer, autumn, and winter, respectively). In addition to data obtained from EPAGRI/CIRAM, wind velocity, temperature of the air and water, and dissolved oxygen (DO) were measured *in situ* during the study. Wind velocity and air temperature were measured with an Instrutherm TAD-500 anemometer, and water temperature (WT) and DO with a YSI 85 multi-parameter probe.

Water aliquots were taken to the laboratory (located at the park), where alkalinity (accuracy of 0.02) and pH (accuracy of 0.01) were measured. CO_2_ concentrations were estimated from measurements of pH and alkalinity (Stumm and Morgan, 1996), with corrections for temperature, altitude, and ionic strength (Cole et al., 1994). Five-hundred milliliters of water was filtered with AP40 Millipore glass fiber filters prior to measuring Chl-*a* after its extraction with acetone (Lorenzen, 1967; Strickland and Parsons, 1972). Dissolved organic carbon (DOC) was analyzed from the filtrate fraction through oxidation under high temperature (680°C) using the Shimadzu TOC-5000 carbon analyzer (Sugimura and Suzuki, 1988).

### Planktonic prokaryotes

Fifteen milliliters water aliquots were fixed with a solution of 2% PFA (para-formaldehyde, final concentration) for further estimation of the abundance, biovolume and biomass of aerobic heterotrophic bacteria (HB) (hereafter HB), and cyanobacteria.

One milliliter aliquots were filtered in dark-polycarbonate membrane filters (θ 25 mm, 0.2 μm) (Millipore), stained with DAPI (4,6-diamidino-2-phenylindole) (1 μg mL^−1^) (Porter and Feig, 1980), and mounted on microscope slides. Bacteria were counted using an epifluorescence microscope (Olympus BX-40) equipped with ultraviolet and green light filters. Ten fields of view were counted from each filter, where the length, width, and elongation of at least 200 heterotrophic bacterial cells were measured. For cyanobacteria, all coccoid cells and filaments were measured. Cell counting and measurements were performed with the “UTHSCSA Image Tool” freeware (University of Texas Health Science Center, San Antonio, TX, USA). Biovolume and biomass estimations were calculated using the algorithm from Norland (1993) and Massana et al. (1997).

### Statistical analyses

Descriptive statistics were used to calculate the minimum, maximum, mean, and standard deviation of data. Log-transformed data showed significant Gaussian distribution (Kolmogorov–Smirnov, *p* < 0.05), homogeneity of variances (Bartlett, *p* > 0.05), and significant matching (*F* test, *p* < 0.05). We conducted one-way ANOVA tests with wind velocity, temperature, pH, DO, Chl-*a*, CO_2_, DOC, bacterial abundance, and biomass for different periods of the day (morning, afternoon, and night), and for different months (October/2009, March, May, and August of 2010). The analyses were followed by *post hoc* testing of Tukey-HSD for multiple comparisons (significance level at *p* < 0.05) (Zar, 1996). Multiple regression models were used to identify the main drivers of CO_2_ and DOC concentrations in the lake. Principal component analysis (PCA) was also conducted using all of the time interval data (daily and between months) and the abiotic and biotic variables (wind, WT, precipitation, DO, DOC, CO_2_, and bacterial biomass – heterotrophic and cyanobacteria). All statistics were conducted using Statistica 7.0 software (Stat Soft Inc., USA).

## Results

### Environmental variables

**Figure 2:**
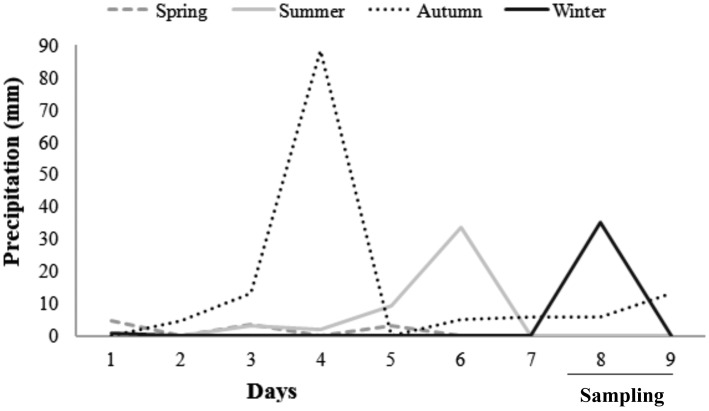
**Precipitation measurements (in mm) for the 7-day period prior to and during the 48-h samplings in October/2009, March/2010, May/2010, and August/2010 in Peri Lake**.

Precipitation in the region of Peri Lake varied from 0 to 90 mm. The maximum value of 90 mm was measured in May, 3 days prior to the sampling (Figure 2). Wind speed was higher in August compared to the other months (ANOVA, Tukey-HSD, df = 48, *p* < 0.05), and significant daily variability was observed in the same period, with winds of 8.5 m s^−1^ in the morning. WT was significantly higher in March, a summer month in the sampling (ANOVA, Tukey-HSD, df = 48, *p* < 0.05), with warmer waters in the afternoon (Table 1).

**Table 1:** **Mean and standard deviation values of wind speed (WS) (m s^−^^1^) measured in the field, water temperature (WT) (°C), pH, dissolved oxygen (DO) (mg L^−^^1^), dissolved inorganic carbon (DIC) (μM), and chlorophyll *a* (Chl-*a*) (μg L^−^^1^) during morning, afternoon, and night hours throughout the 48 h samplings in spring, summer, autumn, and winter**.

Samplings	Period	WS m s^−1^	WT	pH	DO mg L^−1^	Chl-*a* μg L^−1^
October 2009	Morning	2.7 ± 2.5	21.4 ± 1.1	6.3 ± 0.2	9.3 ± 0.6	5.8 ± 0.8
	Afternoon	2.5 ± 1.0	22.0 ± 0.1	6.4 ± 0.1	9.3 ± 0.2	6.3 ± 0.5
	Night	3.0 ± 2.4	20.0 ± 0.1*	6.3 ± 0.1	8.6 ± 0.3*	6.3 ± 1.2
		^b^	^b^*	^c^	^a^*	^b^
March 2010	Morning	1.5 ± 0.2	27.4 ± 1.1	7.5 ± 0.2	7.6 ± 0.4	8.1 ± 0.5
	Afternoon	1.3 ± 0.8	27.7 ± 1.1	7.6 ± 0.2	8.0 ± 0.2	9.3 ± 1.4
	Night	1.0 ± 0.9	26.5 ± 0.5	7.5 ± 0.3	7.4 ± 0.5	8.9 ± 1.3
		^b^	^a^	^a^	^c^	^a^
May 2010	Morning	1.5 ± 1.8	20.7 ± 0.5	7.1 ± 0.1	8.5 ± 0.3	4.9 ± 1.7
	Afternoon	1.9 ± 1.4	20.5 ± 0.7	7.3 ± 0.04*	8.9 ± 0.1	4.2 ± 1.7
	Night	0.7 ± 1.3	20.2 ± 0.4	7.1 ± 0.1	8.3 ± 0.2	5.6 ± 2.6
		^b^	^b^	^b^*	^b^	^b^
August 2010	Morning	8.5 ± 1.8*	17.4 ± 1.4	7.1 ± 0.07	9.3 ± 0.2	8.9 ± 1.5
	Afternoon	4.5 ± 1.6	18.6 ± 1.6	7.2 ± 0.07	9.2 ± 0.2	8.7 ± 0.1
	Night	5.7 ± 0.6	16.6 ± 1.3	7.1 ± 0.03	9.1 ± 0.2	7.9 ± 2.1
		^a^*	^c^	^b^	^a^	^a^

*Lower-case letters indicate the result of ANOVA among samplings followed by the *post hoc* test of Tukey of normalized variables (*N* = 13). For each period of the day (morning, afternoon, and night) a *N* = 4 and *N* = 5 was used. The asterisks represent the different group after ANOVA of daily periods followed by the *post hoc* test of Tukey*.

**Figure 3:**
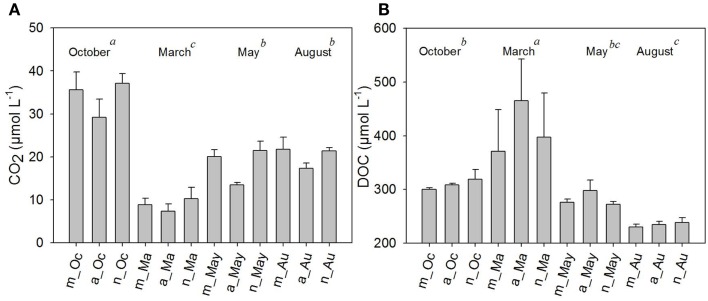
**Average values of carbon dioxide (CO_2_) (A) and dissolved organic carbon (DOC) (B), in μM, measured in the morning, afternoon, and night hours in October/2009, March/2010, May/2010, and August/2010**. Error bars represent the standard errors of each period of the day (m, morning; a, afternoon; n, night) in October (Oc), March (Ma), May (May), and August (Au). Lower-case letters represent the result of ANOVA testing for months followed by the *post hoc* Tukey test for homogenous groups (*p* < 0.05).

The highest pH was also measured in March, and the lowest occurred in October. Similar to temperature observations, pH tended to rise in the afternoon (Table 1). Dissolved oxygen (DO), on the other hand, was higher in August and October (ANOVA *p* < 0.05). There was a small daily shift in DO, with increasing values in the afternoon hours (Table 1).The daily shifts in CO_2_ presented the opposite trend by comparison; whereas WT, DO, and pH increased in the afternoon, CO_2_ decreased in the afternoon. Regarding the annual variability of CO_2_ (between samplings or months), the highest values for both CO_2_ and DO were detected in October and August (ANOVA, *p* < 0.05) (Figure 3A). Chl-*a* showed a daily variation only in March (summer sampling), with increasing Chl-*a* in the afternoon. Chl-*a* was higher during the extreme seasons: summer and winter (ANOVA *p* < 0.05) (Table 1), most likely due to turbulence caused by wind in August (implying the influence of the phytobenthos).

Dissolved organic carbon was approximately twofold higher in March compared to August (ANOVA, *p* < 0.05). No significant diel shifts occurred (Figure 3B); however, a tendency of increasing values in the afternoon compared to morning and night periods was seen in the summer and autumn months (Figure 3B).

### Planktonic prokaryotes

**Figure 4:**
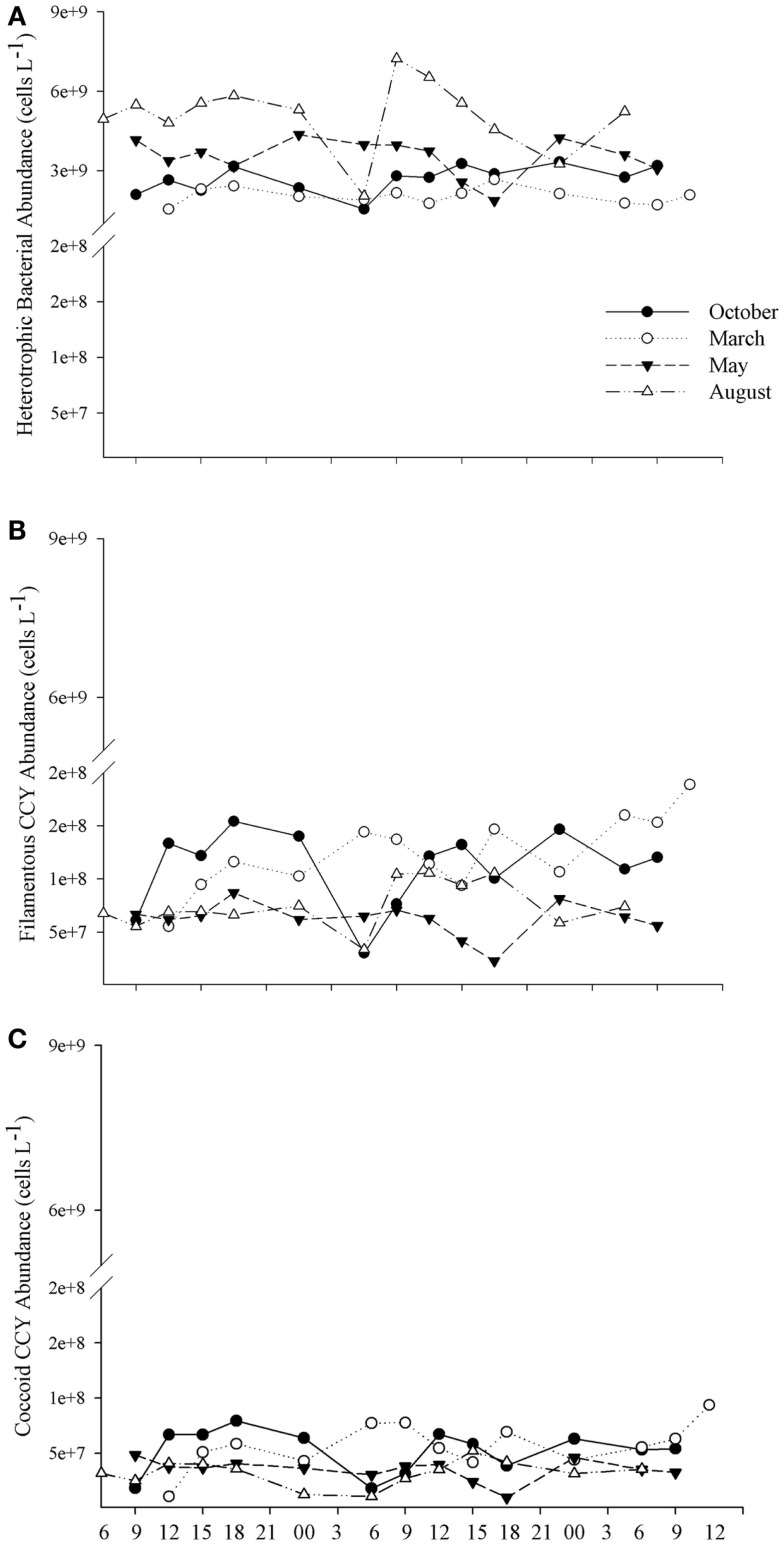
**The daily variability in the abundance of heterotrophic bacteria (A), coccoid cyanobacteria (B), and of filamentous cyanobacteria (C) for each 48 h sampling conducted in October/2009, March/2010, May/2010, and August/2010**.

**Figure 5:**
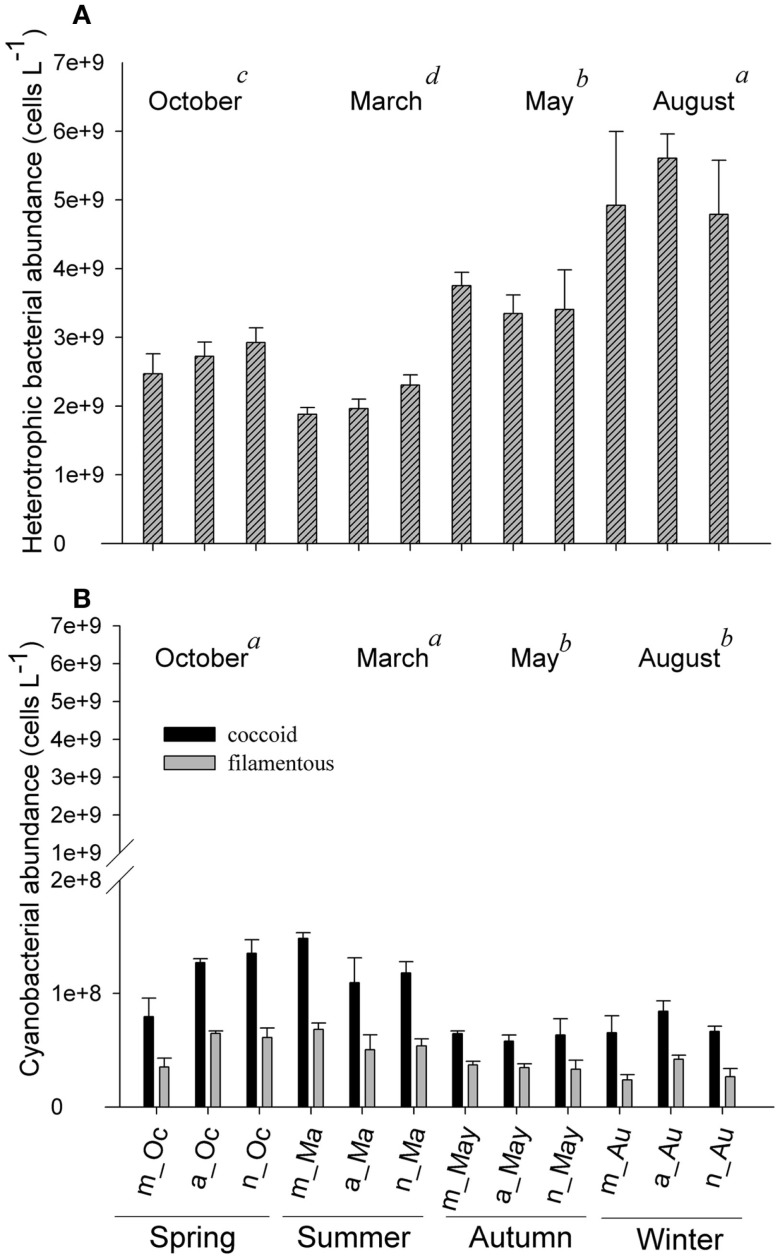
**Fluctuations in heterotrophic bacterial abundance (A) and cyanobacteria (filamentous and coccoid cells) (B), measured in the morning, afternoon, and night hours in October/2009, March/2010, May/2010, and August/2010**. Error bars represent the standard errors of each period of the day (m, morning; a, afternoon; n, night) in October (Oc), March (Ma), May (May), and August (Au). Lower-case letters represent the result of ANOVA testing for months followed by the *post hoc* Tukey test for homogenous groups (*p* < 0.05). The areas shaded gray indicate the periods of lower photoautotrophic abundance.

**Figure 6:**
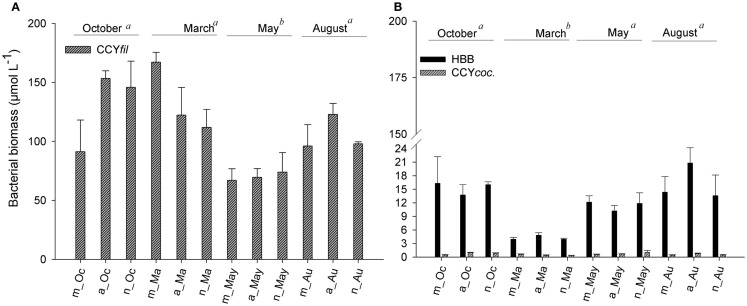
**Average values of biomass of filamentous cyanobacteria (A) and of heterotrophic bacteria and coccoid cyanobacteria (B) measured in the morning, afternoon, and night hours in October/2009, March/2010, May/2010, and August/2010**. Error bars represent the standard errors of each period of the day (m, morning; a, afternoon; n, night) in October (Oc), March (Ma), May (May), and August (Au). Lower-case letters represent the result of ANOVA testing for months followed by the *post hoc* Tukey test for homogenous groups (*p* < 0.05).

In October and March (spring and summer time, respectively), the abundance of cyanobacteria, of both coccus and filamentous cells, was higher in the afternoon than in the morning and night, whereas no increase occurred in May and August (Figures 4B,C). The abundance of the three bacterial groups was minimal near dawn on the first day of October and August, whereas in May, the lowest abundance was observed at dusk (6 p.m.) on the second day (Figure 4). The results show that the density of planktonic prokaryotes changes daily; however, these changes are dependent on day-to-day variability. Instead, the larger shifts in both abundance and biomass of bacteria were detected between samplings (Figures 5 and 6).

Heterotrophic bacteria peaked in the afternoon hours of August (average of 5.60 × 10^9^ cells L^−1^), whereas the lowest average (1.87 × 10^9^ cells L^−1^) was estimated in the morning hours of March, showing a significant variability between samplings (ANOVA, *p* < 0.05). No significant daily variation was observed (ANOVA, *p* > 0.05) (Figure 5A). Biomass varied from 3.95 (March) to 20.83 μmol C L^−1^ (August), with higher values observed in August and October (ANOVA, *p* < 0.05); no daily pattern was observed for the biomass of heterotrophs (Figure 6B).

Filamentous cyanobacteria (CCY*fil*.) present were composed mainly of *Cylindrospermopsis raciborskii* (Woloszynska) Seenayya & Subba Raju (>90%); however, no toxicology study has been published yet to determine the presence of cyanotoxins. CCY*fil*. averages varied from 0.23 × 10^8^ (August) to 0.68 × 10^8^ filaments L^−1^ (March) (Figure 5B). The highest densities of these filamentous cells occurred in October and March, followed by May and August (ANOVA, *p* < 0.05). There was no daily shift in CCY*fil*. abundance (Figure 5B). The biomass of CCY*fil*. varied from 67.00 (May) to 167.16 μmol C L^−1^ (March), with no significant difference between samplings or days (Figure 6A). CCY*fil*. were responsible for the majority of total bacterial carbon (82.3% in the autumn sampling and 97.3% in the summer) in Peri Lake, confirming the importance of filamentous cyanobacteria in concentrating carbon in the form of filaments.

The averages of coccoid cyanobacteria (CCY*coc*.) abundance oscillated between 0.58 × 10^8^ (May) and 1.48 × 10^8^ cells L^−1^ (March) (Figure 5B). CCY*coc*. was higher in March and October, as observed for filamentous cyanobacteria, with no clear daily pattern. However, the biomass of coccoid cyanobacteria varied from 0.38 (March) to 1.07 μmol C L^−1^ (May), without significant variability between samplings (Figure 6B).

The abundance of CCY*coc*. was positively correlated with the abundance of CCY*fil*. (*r* = 0.844, *p* < 0.001), whereas the biomass of CCY*coc*. was positively correlated with the biomass of HB (CCY*coc*. × HB; *r* = 0.427, *p* = 0.002). In addition, there is an excess of DOC in Peri Lake, as carbon in the form of DOC is 18-fold and 100-fold higher than that in the biomass of HB in August and March, respectively (Figures 3B and 6B).

### Multivariate analyses

**Figure 7:**
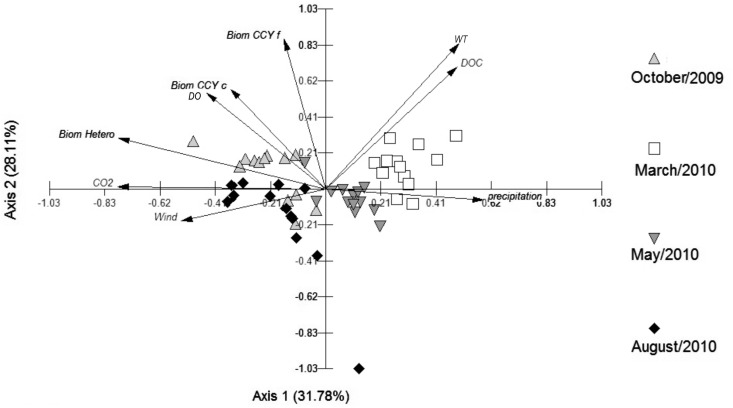
**Principal Component Analysis (PCA) with abiotic and biotic variables (wind, water temperature, precipitation, DO, DOC, CO_2_, and bacterial biomass – heterotrophic and cyanobacteria)**.

The PCA depicted in Figure 7 shows 60% of the data variability. The samplings performed in winter and spring were separated from the summer and autumn samplings by Axis 1, with DOC and temperature serving as important drivers for the separation of the summer sampling, and precipitation for the separation of the autumn one. CO_2_, heterotrophic bacterial biomass, and wind velocity were important for the winter sampling, and biomass of CCY (filamentous and coccus) and DO had a greater influence in the spring (Figure 7).

Multiple regression models using DOC and CO_2_ as dependent variables showed that 44% of the DOC variability was explained by the abundance of total cyanobacteria and of HB (*p* < 0.05) (Table 2). Precipitation, WT, and biomass of HB explained 61% of the CO_2_ variability (*p* < 0.05) (Table 3).

**Table 2:** **Multiple regression model for dissolved organic carbon (dependent variable) using abiotic and biotic independent variables (precipitation, water temperature, dissolved oxygen, wind velocity, chlorophyll *a*, heterotrophic bacterial abundance and biomass, total cyanobacterial abundance and biomass)**.

	Beta	Std. Err.	B	Std. Err.	*t*(45)	*p*-Level
Intercept			2.193	1.765	1.243	0.220
DO	−0.242	0.129	−0.091	0.049	−1.867	0.068
Hetero Ab.	−0.375	0.122	−0.245	0.079	−3.080	**0.003**
Total CCY Ab.	0.554	0.220	0.316	0.126	2.519	**0.015**
Total CCY Biom.	−0.297	0.213	−0.143	0.104	−1.375	0.176

*Data were log-transformed in order to achieve normality. *N* = 50, *R*^2^ = 0.4816, adjusted *R*^2^ = 0. 4356, *F*(4, 45) = 10.454, *p* < 0.001. Significance of bold numbers, *p* <0.05*.

**Table 3:** **Multiple regression model for carbon dioxide (dependent variable) using abiotic and biotic independent variables (precipitation, water temperature, dissolved oxygen, dissolved organic carbon, wind velocity, chlorophyll *a*, heterotrophic bacterial abundance and biomass, total cyanobacterial abundance and biomass)**.

	Beta	Std. Err.	B	Std. Err.	*t*(42)	*p*-Level
Intercept			12.471	3.814	3.270	0.002
WT	−0.550	0.177	−0.098	0.032	−3.106	**0.003**
Hetero Biom.	0.314	0.147	2.731	1.283	2.129	**0.039**
Precipitation	−0.345	0.122	−0.041	0.015	−2.819	**0.007**
Wind	−0.261	0.145	−0.063	0.035	−1.803	0.078
DOC	−0.194	0.123	−0.478	0.303	−1.578	0.122
Chl-*a*	−0.129	0.121	−0.228	0.215	−1.062	0.294

*Data were log-transformed in order to achieve normality. *N* = 50, *R*^2^ = 0.6620, adjusted *R*^2^ = 0.6057, *F*(7, 42) = 11.752, *p* < 0.001. Significance of bold numbers, *p* <0.05*.

## Discussion

The annual variability of the abundance and biomass of planktonic prokaryotes, both heterotrophs and cyanobacteria (filamentous and coccoid), was pronounced. Therefore, small daily patterns in the concentrations of DOC, CO_2_, bacterial abundance, and biomass were observed. Day-to-day variability was reported for metabolic rates (net ecosystem production, gross primary production, and respiration), and changes in available incident irradiance was the main explanation for such variations (Staehr and Sand-Jensen, 2007).

Despite daily shifts in CO_2_ not being significant, a trend of decreasing concentrations in the afternoon compared to morning and night was observed. This trend was contrary to temperature, pH, and DO patterns, which were not observed in the annual scale. These opposing findings suggest that biological constraints are more pronounced in smaller scales (at the diel scale as opposed to the annual scale). This is because oxygenic photosynthesis usually promotes a small rise in pH, as CO_2_ is assimilated (thus, reducing the number of protons in the water). As oxygenic photosynthesis is stimulated under summer light intensities (Tonetta et al., submitted), the higher amount of solar radiation in the afternoon, when compared to morning hours (on a diel scale), could have stimulated primary production during this period of the day.

Additionally, the increasing abundance of cyanobacteria in the afternoon hours reinforced the suggestion that they downregulate CO_2_ in the afternoon. At night and in the morning, CO_2_ concentrations were higher. Accordingly, Sadro et al. (2011) reported a diel variability in planktonic community respiration in Emerald Lake, where higher aerobic respiration rates occurred at dusk and in the first part of the night. In Peri Lake, the higher concentrations of CO_2_ in the morning and at night were observed in all samplings.

Therefore, the variability of CO_2_ between months of sampling was significant and much higher than the daily shifts. The PCA graph showed that temperature, precipitation, and DOC were the variables that were positively correlated with the summer month, whereas HB, wind, and CO_2_ were higher in the winter. Thus, changes in the physical-chemical and biological factors are more pronounced annually than at the 48-h scale. In general, precipitation, temperature, and biomass of HB played the main role in the regulation of CO_2_ dynamics (Table 3). However, precipitation and temperature were inversely correlated with CO_2_. As higher temperature results in decreasing the solubility of gases dissolved in the water, loss of CO_2_ would be expected in the summer months, when WT reached an average of 27°C, compared to 17°C in winter. However, this was not the case. Loss of CO_2_ in temperate lakes is higher in winter and incorporation higher in summertime (Trolle et al., 2012). In Peri Lake, CO_2_ fluxes between the atmosphere and the water were negative in the summer (creating an atmospheric CO_2_ sink) and positive during other periods (Tonetta et al., in preparation). These observations point to the importance of biological factors in carbon dynamics within lakes.

In shallow coastal systems, wind plays a role in stimulating turbulent processes, which increase sediment resuspension and further release DOC and CO_2_ from carbon-rich sediments and pore-water. However, in this study, the lack of a relationship between wind × CO_2_ and wind × DOC, demonstrated by the multiple regression model, suggests either that the sediment is not an important source of carbon to the water column or that wind velocity was not strong enough to increase the upward release. Another explanation is the frequency of carbon measurements (within 48 h), as bacterioplankton abundance respond to turbulence after approximately 6–8 days of turbulence (Arin et al., 2002). Furthermore, DOC was higher in the calm weather periods, supporting the importance of cyanobacteria to organic carbon release.

Carbon dynamics in lakes is very difficult to model, as the terrestrial inputs and mineralization are difficult to measure (Hanson et al., 2011). The authors of that study suggested a few easily measurable parameters to estimate the fate of DOC in lakes, such as lake morphometry, residence time, and temperature, when the recalcitrance of DOC is known. As Peri Lake is a large, shallow lake, according to the Hanson et al. (2011) model, the residence time might exceed 1 year, and the majority of carbon may be lost through mineralization. This points to the importance of heterotrophic planktonic prokaryotes to the metabolism of the lake, which is corroborated by the fact the lake is net heterotrophic on an annual average (Tonetta in preparation).

Interestingly, DOC was higher in the summer and was dependent on cyanobacterial abundance, as shown in the multiple regression model. CCY, the dominant phytoplankton in Peri Lake (Hennemann and Petrucio, 2011), were positively correlated with DOC. This relationship indicates the autochthonous production of DOC by cyanobacteria, which was more pronounced in the summer. Cyanobacteria are, consequently, important producers of bacterial biomass and of DOC in Peri Lake, especially in the afternoon hours of the summer and spring months. Actively growing algae release substantial amounts of DOC via photosynthetic extracellular release (PER) (Baines and Pace, 1991). This exudation of organic material occurs when algal carbon fixation exceeds synthesis of new cell material during periods of sufficient irradiance (Panzenböck, 2007) and under nutrient-depleted conditions (Berman-Frank and Dubinsky, 1999). However, PER of organic matter by intact phytoplankton cells is not the principal pathway for DOC uptake by HB, but instead, byproducts of animal ingestion and digestion are (Jumars et al., 1989; Saba et al., 2011). Zooplankton feeding strategies may also be important to the production of DOC in Peri lake waters, but such were not evaluated in this study.

Terrestrial organic carbon subsiding CO_2_ emissions through community respiration have been reported in other lakes around the world (Cole et al., 2006, 2007; Marotta et al., 2012). Consequently, the terrestrial dissolved organic carbon (tDOC) may account for up to 76% of pelagic bacterial demand (Cole et al., 2006). However, less than just 2% of the bacterial carbon was estimated to be transferred to zooplankton and, thus, up to the food web (Cole et al., 2006). This process results in the sink of carbon in the lake.

Comparing 151 temperate lakes, Trolle et al. (2012) reported the highest efflux of CO_2_ in winter and in those lakes with low Chl-*a* (<11.2 μg L^−1^), demonstrating a link with the trophic state of lakes and CO_2_ flux. As Peri Lake had a Chl-*a* concentration of 7 μg L^−1^ during this study, it might emit CO_2_ to the atmosphere. This was already confirmed by previous studies regarding the CO_2_ fluxes across two time scales: a 1- and a 5-year period (Tonetta et al., in preparation; Fontes et al., in preparation).

Heterotrophic bacterial abundance increased significantly in the winter in Peri Lake, most likely because HB are not regulated by availability of light as cyanobacteria are (Sarmento, 2012). In addition, the mixing processes caused by wind action over the surface of the lake improve the resuspension of organic material, indirectly stimulating HB. Irradiance has been reported to be the main factor shaping planktonic prokaryotes structure in a stratified subtropical lagoon, whereas the activity of cyanobacteria was stimulated in the bottom waters only given available light (Fontes and Abreu, 2009). Heterotrophic bacterial biomass was also higher in August (winter), indicating that heterotrophic bacterial production was stimulated. On the other hand, the abundance and biomass of cyanobacteria were higher in the warmer periods (spring and summer).

In Peri Lake, most of the bacterial carbon was stored in the filamentous cyanobacteria (mainly *Cylindrospermopsis raciborskii*), encompassing up to 98% of total bacterial carbon in March of 2010. This large reservoir of carbon is due to their larger cell size and filament forming strategy, which can function as a predation avoidance mechanism (Bouvy et al., 2001; Pernthaler et al., 2004). The abundance of coccoid cyanobacteria, or picophytoplankton, followed the filamentous bacteria pattern, as observed in the Conceição Lagoon (Fontes and Abreu, 2009), whereas their biomass followed the trends observed for the biomass of HB. However, DOC was produced by both groups of cyanobacteria, as shown in the increase in the abundance of total cyanobacteria. As *Cylindrospermopsis* is the dominant phylogenetic group of phytoplankton in Peri Lake, prokaryotic phytoplankton is important to production of particulate and dissolved carbon there as well.

With global warming, it is estimated that the levels of cyanobacteria will increase in shallow lakes (Kosten et al., 2012) and that more intensive rainfall events will occur, resulting in the intensification of lake CO_2_ emissions (Cole et al., 2006; Dodds and Cole, 2007; Marotta et al., 2009a, 2010a,b). In addition, increasing organic material discharged into the water body might stimulate even greater respiration rates (Del Giorgio et al., 1997; Cole et al., 2006; Staehr et al., 2012). Our results indicated a direct relationship between CO_2_ concentrations and the biomass of HB, pointing to HB as important players in lake community respiration. We showed that smaller and more abundant HB were more important in winter, whereas larger and less abundant filamentous cyanobacteria predominated in summer.

Changes in the community structure of planktonic prokaryotes over 48 h were small, but a general trend was observed in the spring and summer samplings, with an incremental increase in the abundance of coccoid and filamentous cyanobacteria in the afternoon. Significant changes in the abundance and biomass of planktonic prokaryotes and dissolved carbon (DOC and CO_2_) were observed in the annual scale, when the CO_2_ concentration and biomass of HB increased during colder and drier periods. Cyanobacteria, especially filamentous cyanobacteria, produced the majority of bacterial biomass and played an important role in releasing DOC into the water column, particularly in the summer. Thus, planktonic prokaryotes may play an important role in the dynamics of both dissolved inorganic and organic carbon in the lake.

## Conflict of Interest Statement

The authors declare that the research was conducted in the absence of any commercial or financial relationships that could be construed as a potential conflict of interest.
